# The challenges of co-extraction of animal and plant proteins from transgenic plants for use in food and feed

**DOI:** 10.3389/fpls.2025.1626856

**Published:** 2025-08-26

**Authors:** William R. Aimutis, Rohan A. Shirwaiker

**Affiliations:** Bezos Center for Sustainable Protein at NC State, North Carolina State University, Raleigh, NC, United States

**Keywords:** protein, molecular farming, muscle, animal-based proteins, leaf proteins, protein concentrates and isolates, plant-based, extraction

## Abstract

Growing consumer awareness about health, environment, and animal welfare has pressured the food industry to be less reliant on animal proteins consumed as a whole product or formulated into a variety of foods. While recognizing the benefits of complete animal proteins, consumers are increasingly adding plant-based meat-, dairy-, seafood-, and egg-alternatives to diversify their diets. However, these alternatives still lack quality, flavor, and textural characteristics animal protein consumers are accustomed to. The challenges in producing affordable, sensorily acceptable plant-based protein products begin at harvest and in the initial extraction processes. This review highlights the current state-of-the-art in plant protein extraction and then relates these to potential challenges and opportunities in molecular farming wherein animal genes are inserted into plants to produce animal proteins. Plant protein quality is influenced by plant characteristics, environmental and climatic influences, harvesting, and the initial extraction steps. Many of these steps are well understood by actors across the food supply chain. As society begins preparing for large increases in protein demand over the next two decades, molecular farming has the potential to create novel protein offerings with higher nutritional quality, especially when the animal proteins are co-extracted with plant proteins, to meet consumer expectations. Bio-chemical/pharma industries have pursued animal protein extraction from transgenic plants for three decades, but efforts to produce food protein concentrates and isolates containing both animal and plant proteins are nascent, with most work accomplished in laboratories. We propose considerations to progress this technology from laboratories to commercial scale and highlight the importance of communication and education across the food supply chain, including regulators and policy makers, for acceptance and success of these novel products. There will undoubtedly be resistance, but perseverance to answer many questions needs to be recognized in preparation for meeting the rapid protein demand.

## Introduction and background

Alternatives to raising livestock for producing meat, dairy, and egg proteins for human consumption are being promoted to save our planet. The environmental cost of livestock farming is 70% of our agricultural land, 25% of our freshwater footprint, 40% of arable cropland, and a consequential generation of over 70% of food-related greenhouse gas emissions (GHGe) (Der Heijden et al., 2023; Ornan and Reifen, 2022; Ritchie et al., 2019; Silva et al., 2020). Other negative consequences of intensive livestock production are regional biodiversity losses, water eutrophication from manure and urine runoff, and soil acidification. Animal proteins as macronutrients uniquely provide essential amino acids and other micronutrients including vitamins and minerals to sustain human life and are more complete than plant-based proteins (PBP). However, the global population will consume 67% more protein per capita causing our global supply chain to require 40% additional protein by 2050 (FAO, 2009). This prediction nearly two decades ago has encouraged the food industry to develop additional means to provide protein to human diets in the future. Recent evidence indicates continued environmental changes and continued growth of an aging population necessitates healthier and more environmentally favorable foods (Smith et al., 2024; van Vuuren et al., 2025).

While combinations of PBP can fulfill these needs, societal norms have imprinted consumers to appreciate animal-based proteins more readily. There are several questions mankind must solve in coming decades. First, how do we feed 10 billion people in 2050 the proteins needed to sustain life? Also, where will these proteins be derived from both figuratively and geographically? Technology is supplying choices albeit some less favorable than others. For example, plant-based proteins are frequently used nowadays to provide consumers with dairy, meat, egg, and seafood alternatives. These products have gained some acceptance, and their environmental impact is less damaging than traditional livestock agriculture. Other emerging alternative proteins are derived from algae, fungi, yeast, and insects, although food neophobia has tainted these products from widespread consumer acceptance. How do environmental, food, and medical professionals convince animal-based protein (ABP) consumers to change their cultural habits to save our planet? Is molecular farming (MF) of ABP a regulatory and consumer acceptable technology to mitigate environmental harm?

Animal-based proteins can be expressed in plants by insertion of animal DNA into the plant’s genome (Tschofen et al., 2016). Transgenic plants are usually propagated and grown in controlled environment agriculture facilities (CEA), in bioreactors if using plant cell lines, or outdoors for whole plant cultivation in secured fields protected by land buffers on all sides. MF originated in 1989 to produce recombinant proteins used as pharmaceuticals and eventually non-pharmaceutical products including enzymes and personal care biochemicals (Hiatt et al., 1989). The biopharma industry has been using transgenic plants to produce vaccines and monoclonal antibodies for the past three decades (Hiatt et al., 1989; Sijmons et al., 1990). Most recently there is growing momentum to use biotechnology tools in crops to produce ABP in a sustainable and resource-efficient manner (Tusé et al., 2024). There are benefits for using MF in terms of costs, scalability, safety, and perhaps consumer and regulatory acceptance. Although some knowledge gaps still exist regarding choice of target proteins and nutritional equivalency, these products should face less regulatory and legislative scrutiny than cell cultivated meat and seafood alternative products. Recent disruption in the egg supply chain caused by avian influenza and its possible zoonotic risk of crossing over to other species, including humans and cattle (dairy and beef), makes a significant case for promoting this technology. Many of these benefits were highlighted by Tusé et al. (2024) and not reiterated here.

Many commercialized food ingredients (e.g., oils and starches) today are derived from crops such as soybeans, corn, canola, and wheat, which have traditionally been grown to feed livestock for meat and dairy production (Sari et al., 2015). Often after primary products are extracted, residual meals are further processed to manufacture PBP. However, the harsh methods used in primary and secondary extraction steps are not always best for protein yields and quality (González-Pérez and Vereijken, 2007; Zheng et al., 2014). There is parallelism in the challenges of extracting proteins from MF plants to PBP extraction from traditional farming operations. There are only a few companies using MF to produce very limited quantities of common dietary ABP, and only a fraction of those are growing their crops outdoors. These numbers will undoubtedly grow as consumers accept MF as a technology to provide future protein needs. Increased demand for dairy, meat, and egg protein will encourage additional companies to move their growing operations outdoors enabling more acreage to be planted. While upstream genetic insertion and growing methods have become routine, downstream processing will be challenging. The eloquent separation technologies used in a laboratory will not allow mass protein extraction and concentration from field-grown transgenic plants yielding tons of biomass. Furthermore, these methods are time consuming and expensive in a commodity-based manufacturing theatre. Historically, protein extraction from botanical materials is a difficult task often complicated by the presence of other micro- and macro-nutrients. In downstream processing of MF materials, initial processes used to reduce raw agricultural materials (e.g., leaves, stems, seeds, roots) from macro- to micro-scale are critical for successful protein extraction, yet these steps have received minimal attention in MF plant protein extraction literature. These processes are considerably different from those described in the broader MF literature (Buyel et al., 2015).

This review first discusses plant protein extraction, especially highlighting the many challenges beginning at harvest and in the initial extraction steps that ultimately cause defects and failures in the formulated product. The goal is to relate this knowledge to the potential challenges to be encountered when ABP genes are inserted into plants. We focus on the primary extraction steps as they are most pertinent from a plant science perspective, and they also set the stage for final product quality. We offer a perspective on using traditional farming methods for transgenic plants with an intent to produce commercial quantities of hybrid animal/plant protein concentrates and isolates. Topics covered include basic information about the role of physical structure in directing process parameters historically used for other botanical ingredients of economic importance from these crops and now extended into initial protein extraction. We provide a short commentary about persistent challenges after initial extraction that ultimately impact finished products. We will not discuss established and emerging methods to assist in extraction; these are extensively reviewed elsewhere (Ahmed et al., 2024; Anoop et al., 2022; Jha and Sit, 2022; Kumar et al., 2022, 2021), and many are not fully scaled for commercial production yet. Finally, thoughts on strategies and research ideas to overcome challenges detailed in this paper will be presented.

## Plants as food sources

Food ingredients have been extracted from plant-based materials for thousands of years, starting with grains from rice, wheat, and barley. Progressive improvements in food processing technology have further capitalized on extracting value-added phytochemical components for commercialization. Plants are very efficient metabolic machines for production of macro- and micro-nutrients. The macronutrient, protein, was first commercially extracted nearly 75 years ago, and in recent times has gained prominence in our daily food supply. Consumer awareness about healthy eating, environmental effects, and ethical animal treatment has pressured the food industry to be less reliant on ABP whether consumed as a whole food or formulated into food varieties. The growing recognition that animal meat is healthy when consumed in moderation to maximize nutritional value and limit the negative aspects (e.g. cholesterol) has contributed to the emergence of plant-based meat-, dairy-, seafood-, and egg-alternatives. These PBP alternatives, however, are yet to achieve the quality, flavor, and textural characteristics culturally imprinted on consumers’ desires. Production of affordable, healthy and tasty PBP products is dependent on many variables including Mother Nature. While farmers and protein manufacturers cannot control their climatic conditions, they should work together to mitigate other variables impacting PBP quality.

Cutting-edge biopharma tools are being deployed for producing foods of the future. For example, precision fermentation and cell cultivation are using protein engineering tools to provide alternatives to meat, egg, and dairy products. This industry is early in their journey to producing the massive quantities of alternative proteins affordably to feed a growing global population. To build a substantial alternative protein supply chain, several entrepreneurial companies are using protein engineering tools in combination with traditional farming to cultivate plants to synthesize ABP in their various tissues for subsequent extraction and concentration. Furthermore, technology has progressed for scientists to select promoters that target expression within plant-selected systems or cell organelles to maximize protein synthesis (Larrick and Thomas, 2001; Turetta et al., 2025).

Crop selection for transgenic production of food micro- and macro-nutrients is a controversial topic. The biopharma industry has extensively used tobacco and food crops like cereals and legumes as hosts to insert genes (Peters et al., 2013; Rybicki, 2009). However, a deep-rooted agricultural infrastructure has ordained traditional cereal crops (e.g., corn, rice, barley, soybeans) not be used for producing recombinant proteins used as therapeutics and industrial chemicals (e.g., enzymes). Decades of genetic characterization and manipulation have simplified the ability to maximize expressed yields of desired plant compounds. Furthermore, traits for environmental tolerance, pesticide- and predator-resistance, and harvestability have accelerated development and commercialization times when novel genes are inserted for targeted therapeutic molecules. However, the MF concept was limited for many years to CEA or in bioreactors as cell or organelle suspensions while global regulatory agencies scrutinized if transgenic plants should be authorized for outdoor growth by traditional agricultural practices. Anxiety also exists about using crops fed to large populations should there be enhanced expression of allergenic or anti-nutritional phytochemicals (Klocko, 2022). As biotechnology tools further advanced, the United States FDA released a guidance document clarifying the regulatory approach for assessing safety of foods intended for human or animal consumption derived from genomically modified plants (FDA, 2024). The potential for a known food allergen to be transferred from one food source to another remains a concern, and it is highly recommended new entrants in these areas consult with regulatory officials early and frequently in the development process.

The few emerging companies producing MF food proteins have been raising their crops in CEA facilities, and only recently even fewer companies have moved to traditional open-field agriculture. The latter method will be required for future MF to meet the growing protein demand. At current compounded annual growth rates, and with more of the global population consuming increasing amounts of meat protein, we will need over 550 billion kgs of meat by 2050. Although traditional animal agriculture associations publicly stated they will be able to meet this demand, it is unlikely given environmental deterioration and disappearance of farmable acreage (Aimutis and Shirwaiker, 2024). Increased alternative protein production by MF, cell cultivation, PBP, and increased commercial production of other emerging novel proteins need to be escalated over the next two decades to complement animal-produced proteins. We need to start addressing several challenges to biomanufacture these products with safety and nutritional equivalency affordably at scale. Admittedly, cost parity with traditional ABP will be a challenge for some time as companies brave enough to cultivate transgenic plants by traditional farming methods scale-up and optimize manufacturing processes and provide safety evidence. Regulatory agencies will require transgenic crops and reproductive propagules to be contained until more safety data is amassed and intellectually reviewed (Klocko, 2022). However, much of our global population retains an aversion to genetically modified crops and this negativity extends into planting in outdoor fields. A key unanswered question is whether mankind will accept these technologies as food security concerns grow in coming decades and enable bulk production of ABP in common row crops like soybean, corn, potatoes, and possibly wheat. Reality for the foreseeable future is our global population will continue to predominantly depend on traditional agriculture, and new emerging technologies as being discussed here will be supplemental for our needs.

## Role of plant structure in protein synthesis and storage

Plants are physiologically effective at synthesizing proteins throughout their shoot [leaves, stems, and reproductive structures (flowers, fruits, and seeds)] and root systems. Many molecularly expressed indigenous proteins and other phytochemicals within plant cells provide the plant energy from the roots to grain, but others serve unique roles including catalyzing intracellular biochemical reactions or defending against predation. Protein types and yields are genomically expressed by botanical variety but farming practices and environmental conditions during growth also influence these properties (Dupont and Altenbach, 2003). For example, fertilizer and growing temperature affect wheat protein yield and composition (Ciaffi et al., 1996; Wieser and Seilmeier, 1998). Increased protein yield and compositional differences were reported from fertilization and high temperature heat stress in soybeans (Andrade et al., 2023), lentils (Miller et al., 2024), oats (Lee et al., 2023; López et al., 2022) and rice (Liu et al., 2025). In several cases, favorable results were reported for functionality and/or sensory properties under these extreme conditions (Andrade et al., 2023; Liu et al., 2025). Pea protein yields were suppressed in high temperature stress, and elevated levels of heat shock proteins (HSP) are indicative of protein misfolding indicating functional properties are impacted (Devi et al., 2023). Most plants produce HSP in defense of heat and other environmental stresses. As elevated temperatures denature proteins, HSP bind them to protect from misfolding, premature clearance, and aggregation to maintain and restore natural conformations making these proteins still bioavailable to the developing plant (Bukau et al., 2006; Prahlad and Morimoto, 2009). Cultivated chickpeas with elevated levels of HSP, although a drought and heat tolerant crop, are very negatively impacted by prolonged drought and heat stress affecting overall grain yield (Jeffrey et al., 2021; Samineni et al., 2022).

Seed suppliers’ traditional role has been to develop cultivars providing maximum nutrition for targeted consumers (both as animal and human food), and the resulting crops must withstand or tolerate variable pest, climatic, and soil types and conditions including minerality. Plants have evolutionally adapted to the presence of aerosols, atmospheric particles, and other air pollution factors to enhance their ability to absorb solar radiation for photosynthesis and protein expression (Yan et al., 2014). More recently, as plants became important sources for alternative proteins, plant breeders also focused on developing cultivars with reduced synthesis of anti-nutritional factors and volatile organic compound (VOC) precursors that impact flavor. Intense stressful environmental conditions impact VOC formation during cultivation as plants use VOC formation as a defense mechanism (López et al., 2011). Breeders are also selecting cultivars resistant to temperature extremes, insect infestation, and water stress (Vicenti et al., 2019).

**Figure 1:**
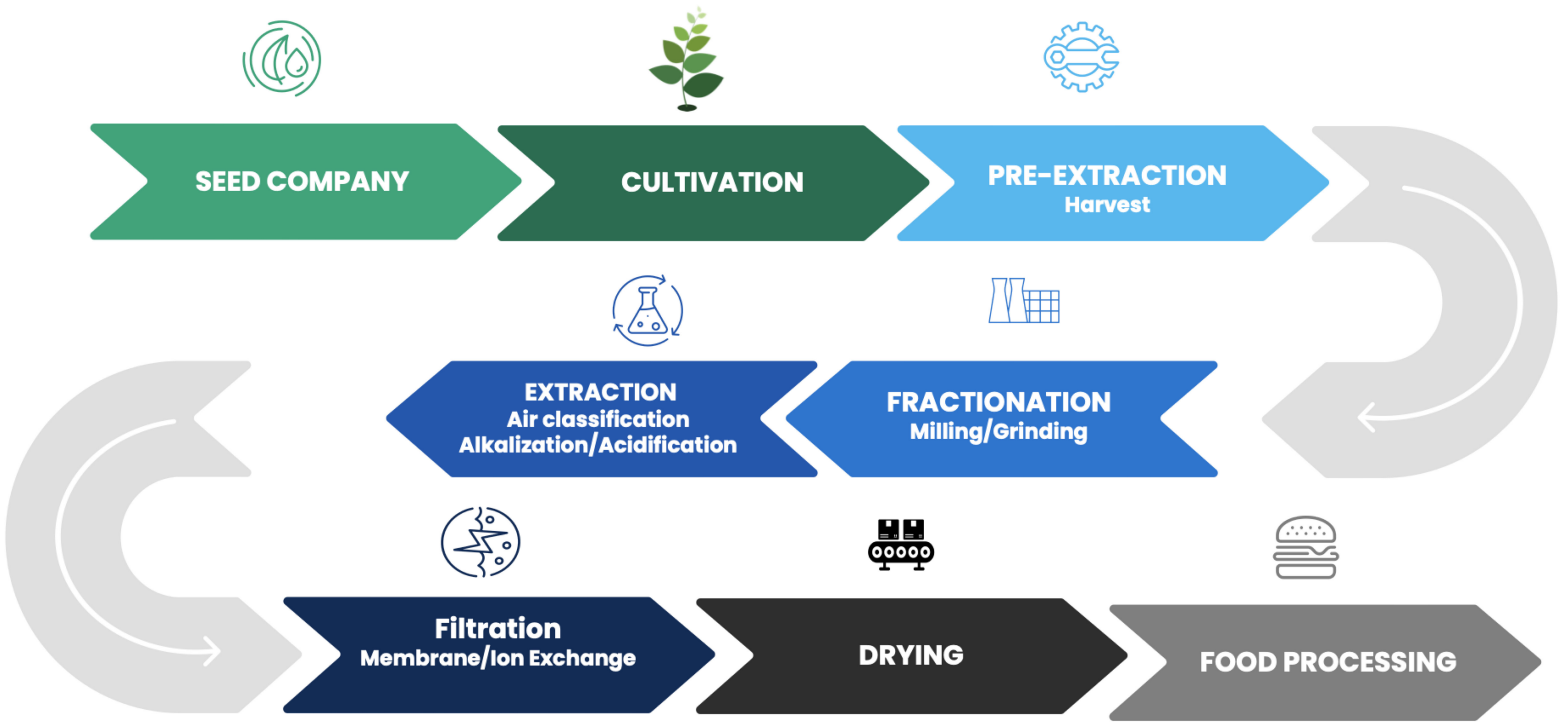
Alternative Protein Manufacturing Flow.

Once seeds are planted, they are in the farmer’s care. Close examination of the agricultural supply chain reveals several instances in the process of moving from a seed to a consumed product that can be erred in producing finished food products to meet consumer demands (**Figure 1**). Nearly every industrial process, especially for food ingredients and products, requires procuring and using the highest quality raw materials possible to ensure final product quality and consistency. Protein extraction, regardless of raw material source, from their native environments is a difficult task often complicated by presence of other micro- and macro-nutrients. Native PBP and transgenic protein extraction are not exceptions (Kumar et al., 2021).

## Downstream processing: extraction methods

Commercial downstream processing is expensive accounting for up to 80% or more of production costs (Menkhaus et al., 2004). Most commercial processes have originated from work done in laboratory environments and subsequently transferred to industrial production with further refinement improving efficiency, often reducing production time and costs. Although most commercial methods are similar and follow suggestions provided by laboratory findings, the reality is not a singular method used with all raw materials provides maximum yield without some negative effects. Multiple trade secret methods exist for protein extraction to produce commercial quantities of protein concentrates (PC) and isolates (PI) (Aimutis and Shirwaiker, 2024). In fact, unique ABP and PBP extraction methods exist solely for extracting the respective proteins, but in combination as we describe in this paper, the task is intensified because of structural and functional differences between ABP and PBP (Day et al., 2022).

Commercial PC and PI are a heterogeneous mixture of dozens of proteins. However, the predominant proteins in selected raw materials account for 80% of the total yield and are responsible for much of the physical functionality of these products. Protein extraction processes involve implementing physical changes or chemical transformations of desired raw material(s) into desired products. For the manufacture of many PBP concentrates (PBPC) and isolates (PBPI), the raw material is typically a byproduct from an initial extraction process for food ingredients such as flours, starches, oils, hydrocolloids, colors, fibers, or other products. They are also byproducts with a mixture of different grains as found in brewer’s spent grains (BSG) or distiller’s dried grains with solubles. There are common challenges in designing a process capable of overcoming a plant’s unique genomically encoded physical structures to liberate protein from freshly harvested crop raw material or using a byproduct from another primary extraction. Several critical factors impact extraction efficiency including extraction equipment and method, plant component matrix properties, extraction solvent, ionic strength, temperature, pressure, and time (Drosou et al., 2015). Selection of physical and chemical extraction methods are decreed by molecular properties of the proteins themselves including their conformation, isoelectric points, denaturation temperatures, and chemical bonding characteristics (**Table 1**).

**Table 1:** Physical properties of major plant-based proteins to leverage in commercial protein extraction^1^.

Source	Protein	Mol. Wgt. (KDa)	# Disulfide bonds/Free thiol groups	Surface hydrophobicity	Protein assembly	pI	Functional properties^2^	T_d_^3^ (°C)	Refs.^4^
Soybean	ß-Conglycinin (7S Globulin)	150-200	0/0	hydrophobic	Trimer: 3 subunits α,α’,and β	5.6-6.0	E,F,G	65-75	Soy
	Glycinin (11S Globulin)	300-380	2/2	hydrophilic	Hexamer: 6 subunits with acidic and basic polypeptides arranged in a hollow oblate cylinder	6-7	E,F,G	85-95	
Yellow Peas	Legumin	320-380	2/1	hydrophobic	Hexamer: 6 subunits with acidic and basic polypeptides	4-5	E,FB,FH,G,T,WB	75-87	Pea
	Vicilin	170	0/0	hydrophilic	Trimer: 3 subunits (47 KDa, 50 KDa, and 34–30 KDa)	4.5	E,F	69-72	
Faba Beans	Legumin	360-400	6/0	hydrophilic	Hexamer: 6 subunits (60 KDa); each subunit 2 polypeptide chains: α-chain (acidic, 40 KDa) and β-chain (basic, 20 KDa) joined by S-S bonds	5.0	E,F,G	95	Faba
	Vicillin	170	0/0	hydrophobic	Trimer: 3 subunits (47 KDa, 50 KDa, 34 KDa, and 30 KDa)	4.8-5.5	E,F,G,FB,WB	83.8	
Oats	3S Globulin	320	2/1	hydrophilic	Dimer: 2 subunits (15 KDa and 21 KDa) joined by S-S bonds	8-9	E,F,G,FB,WB	110	Oat
	7S Globulin	150-200	0/1	hydrophobic	Trimer: 3 subunits (50–70 KDa); held together by noncovalent bonds	8-9	Poor @pH 7	110	
	12S Globulin	320	6/6	hydrophobic	Hexamer: 6 subunits (each 54 KDa); joined by S-S bonds	5.9-7.2	E,F,G	110	
	Avenins (Prolamins)	14-35	4/0	hydrophobic	Usually monomers and dimers in ethanol; primary sequence rich in Pro and Gln	6-9	AO,E,F,G,WB	114-116	
Chickpeas	Legumin (11S globulin)	320-400	6/0	hydrophobic	Hexamer: 6 subunits (54–60 KDa); acidic and basic polypeptides; joined by S-S bonds	4.5	E,F,G,FB,WB	103-107	CP
	Vicillin (7S globulin)	140	0/1	hydrophilic	Trimer: 3 subunits (12.4–67 KDa) linked by noncovalent hydrophobic interactions	4-5	E,F,G	89-107	
Maize (corn)	Prolamins (zein)	25-45	Multiple and varies	hydrophobic	Polymeric oligomers: 4 subunits (α- (19–22 KDa), β(~14 KDa), Ɣ (16,27, and 50 KDa), and δ(10 and 18 KDa)	6.2	E,Ff,T,	58-91	Maize
Rice (brown)	Glutelin	49-65	3-5/1	hydrophobic	Dimer: Acidic (α) subunit (30–40 KDa) and a basic (β) subunit (19–25 KDa); forms large aggregates (100–200 KDa); poor aqueous solubility	4.8	AO,B,E,F,G	73-86	Rice
	Globulin	30-55	4/1	hydrophobic	Hexamer: subunits range in molecular weight from 23–105 KDa	5.9-7.3	E,F	~98	
Barley	Hordeins	12-100	4/2	hydrophilic	Polymorphic: 4 groups (C- (34.3 KDa), Ɣ- (31.4 KDa), B- (33.2 KDa), and D- (72.9 KDa); joined by S-S bonds	6.6	AO,E,F,G,T,WB	~125	Barley
	Glutelin	21-39	Not determined	hydrophilic	Dimer: acidic polypeptide (34–39 KDa) and basic polypeptide (21–23 KDa); poor solubility	5.6	E,F,G,T,WB	~125	

^1^Native molecules aqueous pH 7.0 (except zein which was suspended in aqueous ethanol (70%).

^2^Functionality abbreviations: AO, antioxidant; B, bioactivity; E, emulsifying; F, foaming; FB, fat binding; FH, fat (oil) holding; FeB, iron binding; Ff, film forming; G, gelling; IM, immunomodulatory; OB, oxygen binding; T, texturizing; TP, transport protein; WB, water binding.

^3^T_d_: Thermal denaturation temperature.

^4^References Soy: Hou et al. (2025); Qi et al. (2011); Utsumi and Kinsella (1985); Pea: Lu et al. (2020); Mession et al. (2015); Faba: Oluwajuyitan and Aluko (2025); Feng et al. (2024); Thomsen et al. (2025); Oat: Liu et al. (2009); Li et al. (2025); Schalk et al. (2017); CP: Boukid (2021); Pennells et al. (2024); Soto-Madrid et al. (2022); Maize: Argos et al. (1982); Feng et al. (2021); Esen (1987); Rice: Agboola et al. (2005); Amagliani et al. (2017); Jayaprakash et al. (2022); Kawagoe et al. (2005); Barley: Jaeger et al. (2021); Schalk et al. (2017).

**Figure 2:**
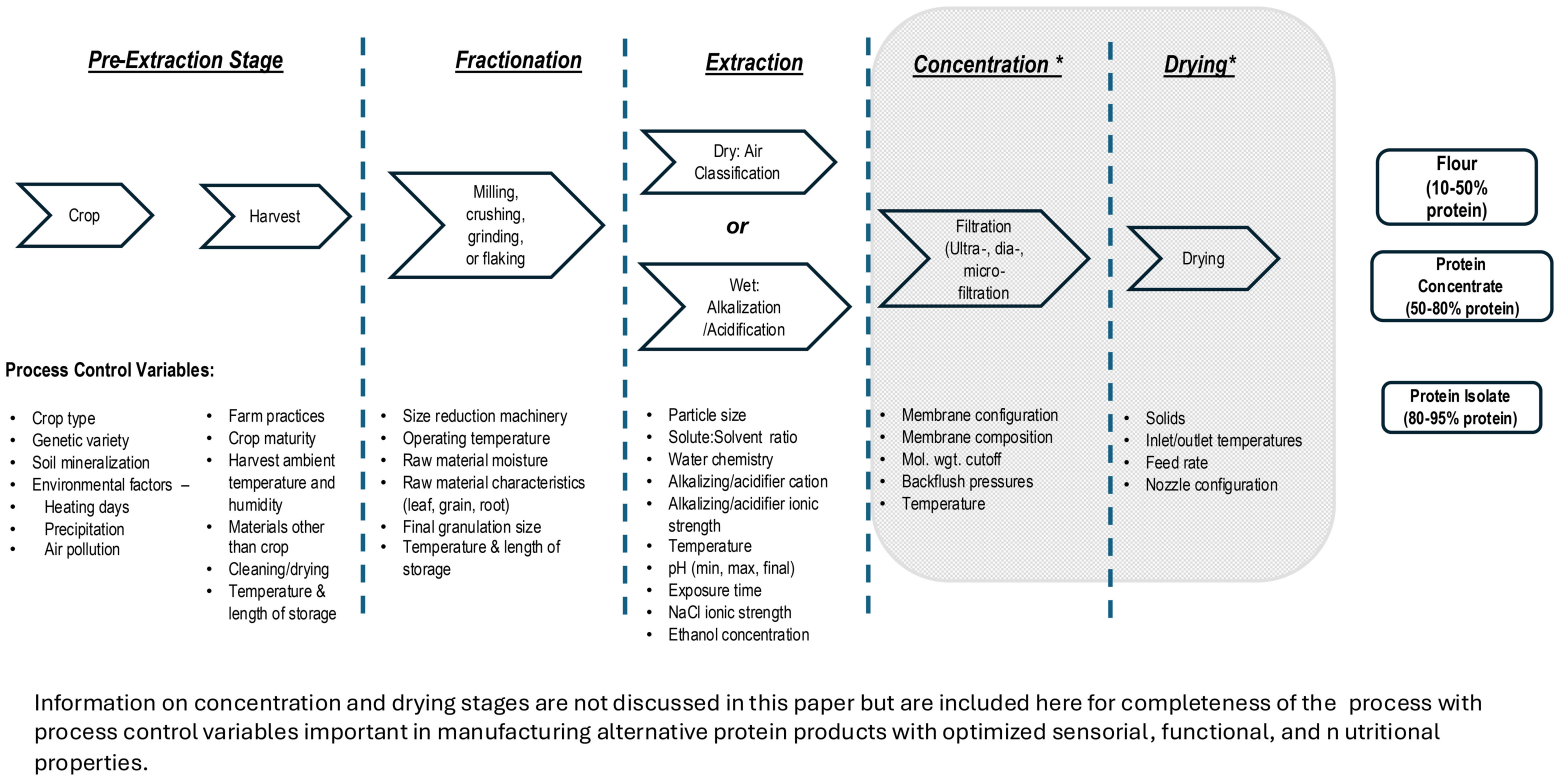
Factors Affecting Alternative Plant-Based Protein Extraction.

An overall schematic of the extraction process along with variables to control is presented in **Figure 2**. Additionally in the schematic, processes beyond extracting proteins from plant materials (e.g., concentration and drying) will not be extensively discussed beyond mentioning them for completeness in manufacture of commercial protein products. Other references on this topic can provide background information to the reader (Gharsallaoui et al., 2007; Lan et al., 2019; Mondor and Hernández-Álvarez, 2022; Strumillo et al., 1995). It is worth noting the nuances of these post-extraction stages are often trade secrets closely guarded by manufacturers.

## Pre-protein extraction: harvest and initial fractionation

In PBP manufacture, the initial steps to procure and store raw agricultural materials prior to protein extraction are the most critical, yet these are minimally discussed in published protein extraction literature. Many challenges and ultimately product defects in PBPC and PBPI originate at this stage (**Figure 2**). Conditions during crop maturation as well as methods used in crop harvesting impact targeted traits and ultimately protein quality. For example, extreme temperatures during crop maturation, fluctuating moisture conditions, and insect and weed infestation, which are marginally controlled by farmers, can negatively impact protein quality and yield. Physical damage by careless harvesting such as leaf shredding, broken seeds and stems, invisible mechanical damage, and materials other than the desired crop (MOC) also adversely impact protein yield and quality when crops are further processed (Mesquita et al., 2006). Skilled farmers are accustomed to good agricultural harvest practices and maintain their equipment in top condition, but sometimes small deviations impact protein quality. For example, combine ground speed and threshing cylinder rotational speed both correlated linearly and positively with increased leaf shredding, broken seeds, seed coat damage, and MOC (Mesquita et al., 2006). Crop moisture levels at harvest impact field losses and plant damage. If root structure is the plant part being pursued, quality deterioration is accelerated by moisture and temperature conditions. Additionally, roots must be processed quickly to avoid protein hydrolysis by endogenous root proteases.

Ideally, plant materials should be immediately frozen upon removal from the field to arrest metabolic reactions initiated by harvesting and forced senescence. Although this is difficult to execute in many farming operations, there are distinct advantages in using this simple technology. The added benefit of freezing at -30° C and thawing at 4° C before extraction is increased protein yield upwards of 20% in subsequent extraction steps (Matsushima and Matsuo, 2024). Plant cells containing expressed recombinant leaf proteins seem especially amenable to this process step, but prolonged frozen storage should be avoided as ice crystal formation critical in rupturing cell membranes to release proteins is also detrimental to protein structure and some desired enzyme activity. The impact of repeated freeze-thaw cycles on protein extraction and ultimately yield and functionality has not been reported, but several studies have shown functionality of PBP and ABP isolates (mung bean and silkworm) is improved by three freeze thaw cycles (Phuangjit et al., 2023; Katekhong and Phuangjit, 2025). This process sequence disorders protein secondary structure causing protein unfolding and exposing sulfhydryl residues resulting in improved foaming and emulsification properties. Increased cycles reversed this effect likely resulting from multiple protein molecules being extensively denatured. Similar results are observed with freeze-thaw cycling of other PBP and ABP concentrate and isolate powders (Feng et al., 2020; Cao et al., 2022; Kumar et al., 2022; Liu et al., 2022). ABP also experience “cold denaturation” because increased solubility of hydrophobic groups enhances protein adsorption promoting aggregation (Dias et al., 2010; Thorat et al., 2020).

## Primary fractionation – raw materials and primary extraction

In absence of freezing, extraction should begin immediately after harvest to ensure minimal damage to protein’s nutritional value and physical functionality (**Figure 2**). This also prevents formation of offensive colors, aromas, and flavors in final commercialized protein products. Endogenous plant proteases and lipases released from harvest-damaged cells begin hydrolyzing native fats and proteins almost immediately. The liberated unsaturated fatty acids and free amino acids are primary contributors to off-flavors (Badjona et al., 2023). Unfortunately, mechanical energy exerted during edible plant tissue harvest invariably leads to cell separation and cell breakage. While not all tissue experiences total failure, a significant amount of cellular damage releases enzymes and other micronutrients, e.g., polyphenols, impacting final aroma and flavor of extracted biomaterials and ultimately PBPC and PBPI. Ultimately, extraction methods must address product quality, process efficiency, and production costs in the most environmentally favorable and socially sustainable manner (Jha and Sit, 2022).

The primary fractionation steps after harvest involve reducing incoming raw materials (seeds, leaves, roots, etc.) to smaller particle sizes to improve extraction efficiency. Milling, grinding, chopping, crushing, and maceration are traditional methods used to reduce crop components to smaller particles. Conventional wisdom indicates smaller particle sizes provide increased surface area for subsequent protein extraction and increased yields; however, this generally causes protein damage. Physical processes involved in milling and maceration potentially generate heat causing protein unfolding and denaturation ultimately affecting nutritional value and protein functionality. Excessive grinding disrupts secondary and tertiary protein structures impacting physical functionality in cereal protein isolates (Elliason and Hegg, 1980; Liu et al., 2021). Ultrafine protein powders typically have reduced protein solubility, but increased emulsion stability (Silventoinen et al., 2021).

*Historical Perspective:* Milling dates to 2600 BC (Walker and Eustace, 2016) when early human discovered they could grind plants, seeds/grains, stems, and roots to smaller fragments making it easier to prepare plant materials as foods. They learned this by pounding and rubbing plant material between stones, and eventually hydro energy-driven milling facilities were built to move much larger stones, theoretically extracting 100% of the raw material into useful flour (Walker and Eustace, 2016; Kihlberg et al., 2004). Reluctantly, stone milling also exerts considerable heat from friction potentially damaging protein, starch, and fatty acids (Prabhasankar and Rao, 2001). Stone milling is currently used for special purpose flours, and stones can often be found in many modern milling plants.

*Mechanization and Modernization:* Roller mills are widely utilized in the food industry for a variety of ingredients. Milling machinery comprises two large co-rotating or counterrotating cylindrical rollers with minimal tolerance in between, exerting mechanical forces capable of crushing grain into smaller fragments and fine particles. Typical flour particle size is 10-300 µm, but up to 10% of the total flour particles are <10 µm. Much of this ultrafine flour only functions as dietary fiber because proteins and starch granules have been damaged by abrasive and thermal energies. While this processing method also generates heat potentially damaging macronutrients, increased thermoenergy on the roller surfaces can be minimized by circulating coolants through the rollers during operation. The ideal operational range for roller mills is 25-65° C. The denaturation temperature range for most PBP is 70-95° C depending on protein source and concentration (**Table 1**). Plant-based starches typically gelatinize at 60-90° C although some crops are higher (e.g., corn, rice, and sorghum). Grain moisture is ideally 14-16% for efficient milling and excellent flour quality. Some grains are washed before milling to remove debris or to enable better moisture content for milling. As industry further optimized their processes, the rollers now have unique surface textures and configurations of multiple rollers. Other milling equipment including hammer, pin, sonic, and colloid mills is frequently used to crush or grind harder-surfaced or specialty grains. Further information on milling equipment and processes is available (Kumar Sharma et al., 2021).

The “dry” milling process focuses on flour production from cereals and legumes. Whole grain flour is commercialized at this step, but flour can be further processed using sieving screens to separate granular fractions by size enabling unique product offerings with diversified proximate composition and functional attributes for bakers and other food formulators (Cheng et al., 2023). Baking industry has championed the technical understanding of milling especially of wheat flours of varying particle sizes to enable unique sweet and savory baked goods.

Oilseeds follow a different path before a meal is usable for further commercial processing. Specifically, oil seeds (e.g., soybean, canola, sunflower) are roller milled or screw pressed to “flake” the grain. Herein, mechanical energy presses oil from the grains, which is subsequently processed into edible oils (Chandraskar and Mohan, 2024). The leftover meal is “defatted” by washing with an organic solvent, predominantly hexane. This defatted meal is commercialized for animal feed or further processed by alternative protein manufacturers into PBPC or PBPI using methods described in proceeding sections.

## Aqueous protein fractionation

Aqueous fractionation processing is environmentally unfavorable because it uses large amounts of water and energy to produce PBPC. Nonetheless, gluten proteins are extracted from corn and wheat using a more intensive “wet” milling process. Grains are crushed into warm water and kneaded to form a dough. The mixing process solubilizes starches into the water leaving a gluten protein rich dough that is further washed and eventually dried and milled to desired particle sizes. Cereal gluten PC have poor solubility and limited functionality (predominantly gluten structures). The product is commercialized as “vital” gluten PC. The decanted starch slurry is dried for commercial starch powders or undergoes a saccharification process using enzymes to make high fructose syrups.

Plant leaves have traditionally been treated as agricultural waste after harvest despite this biomass being a large underutilized PBP raw material. Some farmers compost this green mass for soil conditioning or feed small amounts to their livestock. However, plant leaves are the epicenter for photosynthetic activity essential for protein synthesis in other plant components. Photosynthetic activity provides carbon skeletons and energy (NADPH and ATP) for amino acid mobilization and synthesis in the translation process for protein primary sequence assembly (Anoop et al., 2022). Plant leaves contain ~1-8% fresh weight protein and are high in essential amino acids including Thr, and lower levels of Lys, Met, and Ile (Gerloff et al., 1965; Ghaly and Alkoaik, 2010). Plant leaf protein extraction separates soluble proteins from fibrous leaf skeleton using maceration, pressing, or steeping. Roller mills can exude protein “juice”, but usually maceration is more efficient and best accomplished with a screw press. Fractional mass balance of crudely extracted green leaves produces 50% leaf liquid (~5% solids), 45% fiber (45% solids), and 5% leaf protein “concentrate” (~45% solids) (Balfany et al., 2023). Sonication is also used to extract leaf proteins and improves protein yield (Saha and Deka, 2017; Xu et al., 2017). High intensity ultrasound waves are better at disrupting plant tissue, allow better solvent penetration into the cellular material, and accelerate mass transport processes (Hadidi et al., 2024; Vinatoru, 2001). These methods however are very inefficient, and not at commercial scale to process tons of raw material daily. In addition to removing proteins, numerous other plant molecules are extracted for further separation in later processing steps.

Fifty percent of the soluble protein extracted from green leaves is the CO_2_-fixing enzyme complex ribulose-1,5-bisphosphate carboxylase/oxygenase (Rubisco). Rubisco catalyzes formation of bioavailable sugars from atmospheric CO_2_ and is probably the oldest and most abundant PBP on Earth after genes encoding this protein were transferred from proteobacteria into the red algal chloroplast (Anderson et al., 1968; Prywes et al., 2023). The magnitude of Rubisco is an annual conversion of ~100 gigatons of carbon from CO_2_ to sugar by plants, algae, and cyanobacteria (Bar-On and Milo, 2019). Despite the criticality of Rubisco for carbon assimilation, its enzyme kinetics are relatively slow and limit flux through the carbon fixation cycle to one CO_2_ molecule per turn of the cycle (Jordan and Ogren, 1981).

Rubisco is a quaternary structured protein consisting of 8 small and 8 large subunits (12.5 and 55 KDa, respectively) and is usually located in the chloroplast fraction (Makino et al., 2003). Its functional properties include solubility, emulsifying, gelling, and foaming that closely resemble egg white proteins (Di Stefano et al., 2018; Martin et al., 2014). Furthermore, its gelling properties at low concentration (~2%) and a denaturation temperature like egg albumin (~65° C) to form a firm gel like hen’s eggs make Rubisco a good candidate to create an egg alternative.

This protein is being pursued by several companies for commercialization because of its abundance in nature and unique functionality. However, despite its ubiquity, this protein sequence and conformation is very conserved amongst plant species. Even so, it has been observed that Rubisco’s functional properties unpredictably fluctuate. This molecule is thermodynamically unstable and is constantly conformationally rearranging to recognize and engage in specific interactions with functional, intra- and extra-cellular partners (other proteins, metabolites, nucleic acids, minerals, sugars, etc.) ultimately directing a protein’s function in a process known as *biological recognition* (Fuxreiter, 2025).

Scientists accept protein motions and conformational changes are important in biological work execution and molecules are constantly reshaping to interact with other partners to maintain essential plant functions (Orellana, 2023). Rubisco has been relatively slow to structurally change through evolution but given its biological importance in photosynthesis, maintaining its molecular signature is important to avoid catastrophic mass plant extinction (Barik, 2020).

Root and tuber proteins are mostly involved with active root growth and local environmental interactions. Roots are a warehouse to store nitrogen and excessive amino acids, providing these to the remainder of the plant as needed for *de novo* protein synthesis. In stress situations, plants are triggered to synthesize proteins that counter the stress. Roots maintain plant N balance and if the amino acid pool is depleted, they uptake peptides from their surrounding soil and disassemble them to dipeptides, tripeptides, and free amino acids for transport to other plant regions (Näsholm et al., 2009; Schobert et al., 1988). Soil contains mostly intact proteins that are difficult for plant roots to transport into their cells, and therefore they rely on a symbiotic relationship with soil microorganisms to hydrolyze intact proteins to dipeptides and free amino acids for uptake by the roots (Rentsch et al., 2007; Godlewski and Adamczyk, 2007). Although several roots and tubers are commonly used as human food, they are seldom used for PBPC and PBPI because they have low protein concentrations (1-4%). Furthermore, plants propagated in soils with elevated heavy metal concentrations from natural or contaminated sources tend to store metals in the roots and tubers before circulating to the leaves (Knez et al., 2022; Ye et al., 2020). Although consumption of root vegetables high in Cd has not been demonstrated to cause ill effects, prolonged human exposure to heavy metals eventually accumulates it in tissues and manifests toxic effects (Waqas et al., 2024; Zahra et al., 2017).

Roots grown outdoors in soil have transport mechanisms to uptake heavy metals from their surrounding soil (Ejaz et al., 2023). Whilst some heavy metals in extremely low concentrations may be needed for plant growth, roots can store them until other parts of the plant signal a need for them (Goyal et al., 2020; Hasan et al., 2017). Furthermore, roots are not very efficient at metabolizing and/or clearing heavy metals from their system (Gautam and Agrawal, 2019). While much of the heavy metals may be washed away during protein extraction, there is evidence heavy metals can associate with root and nuclear proteins (Emamverdian et al., 2015). Co-isolation of heavy metal associated proteins could be potentially hazardous if consumed by animals and humans.

## Protein extraction from fractionated plant structures

Consumption of highly concentrated PBP has flourished the past two decades driven by the increasing consumer awareness. The concept of biorefining first appeared in literature in reference to using biomass to produce fuels and industrial organic chemicals (Levy et al., 1981). Eventually this concept was introduced in the food research literature during the late 1990’s when Dr. Mark Etzel at University of Wisconsin introduced biorefining of milk proteins. Today, PBP refining has been discussed in numerous articles (Carpita and McCann, 2020; del Mar Contreras et al., 2019; Muniz et al., 2024; Sari et al., 2015; Xiao et al., 2023; Xiong et al., 2021; Zhao et al., 2014).

The intent at this stage is the removal of unwanted phytochemicals to concentrate proteins from the incoming fractionated biomass (**Figure 2**). This stage most critically affects protein yield and quality, relying on physical properties of solubility and aggregation to concentrate proteins by solid partitioning (Huie, 2002). Several critical elements, including extraction technique, selected solvents, and process temperatures, pressures, and times are combined in a systematic fashion to accomplish protein separation from the crudely extracted milieu (Drosou et al., 2015). It is impractical to extract and isolate all the proteins found in a biomass or plant fraction. Commercial PBPC and PBPI are heterogeneous mixtures that compositionally vary dependent on numerous factors including processing methods used in this extraction stage as well as other accompanying plant substances including carbohydrates and secondary metabolites (Etzbach et al., 2024). Many manufacturers use alkalization and acidification with small nuances to differentiate themselves from competitors to produce commercial PBPC and PBPI. However, these methods are only efficient at removing approximately 50% of the total proteins present in plant biomass (Karki et al., 2010).

Plant structure from seed germination until harvested is highly specialized to compartmentalize, store, and protect proteins until fertilization renews the process (Wood et al., 2011). Highly specialized plant architecture makes protein physical extraction difficult (Burton and Fincher, 2014; Xiong et al., 2021). For example, even though cell membranes are porous to enable movement of nutrients and metabolic by-products into and from the endosperm, PBP extraction from grains or biomass is difficult because cellulose, hemicellulose, and pectin fibers entrap protein molecules in their molecular structure. Electron microscopic examination of plant cell walls shows fiber fractions exist in a highly organized woven format with cellulose and hemicellulose fibers layered upon one another (Carpita and McCann, 2000, 2020). The membrane pores are large enough to allow passage of specific molecules but prohibit protein molecules leaking from plant cells. It is also possible plant cell walls utilize protein molecules as signaling agents or for their own energy sinks. Whatever their physiological role, protein molecules are difficult to extract from their trapped environments even with cellulase, hemicellulase, and pectinase enzymes. Once liberated from physical entanglement, protein extraction and concentration are dependent on the physical actions of aggregation and/or solubilization. Proteins modulate their unique properties based on regional domains of amino acid residues encoded in their primary sequences specifying how the protein molecule folds and associates with other neighboring proteins or compounds (**Table 1**). Most extraction methods disrupt these regions to induce aggregation or solubilization. Protein dynamics are influenced by hydrophobic, hydrophilic, and disulfide regions within each protein molecule (Bulaj, 2005).

## Air classification method

Air classification processing is beneficial for dry fractionation of native proteins from grain endosperm or cotyledons (pulses) in a sustainable manner (Schutyser and van der Goot, 2011). This process is optimal when grains have dried beyond the 14-16% moisture recommended earlier. In fact, increased brittleness is advantageously used to rupture plant tissues with lower energy usage and into smaller particles than achieved by typical milling (Tyler and Panchuk, 1982; Laskowski and Lysiak, 1999). Flour from this first “break” is subsequently ground using pin mills or jet mills to produce an ultra-fine particle flour (Tyler, 1984). These milling methods generate less heat than stone and roller mills yielding flour that has broken the endosperm and cotyledon into very fine fragments without damaging tissue components like starch granules (Boye et al., 2010). Once particles are dispersed into an air classifier, cyclonic air movement aerodynamically separates protein enriched particles from starch- and fiber-enriched particles based on density and particle size (Schutyser and van der Goot, 2011). Air turbulence, particle collisions, and interparticle bridges affect protein yield (Pulivarthi et al., 2023). Optimum separation occurs when particle sizes are ~10 µm to minimize protein/starch interactions (Dijkink and Langelaan, 2002). Often electrostatic separation is combined with air classification to improve classification efficiency. Electrostatic separation classifies particles by applying electrical forces to particle surfaces. This technology is used to produce highly enriched aleurone flour from wheat and barley (Bohm et al., 2011; Laux et al., 2006). The aleurone fraction is a bioactive component enriched in protein (~15-17%) and antioxidants beneficial in intestinal health and inflammation reduction (Brouns et al., 2012).

## Protein extraction using solvents

**Figure 3:**
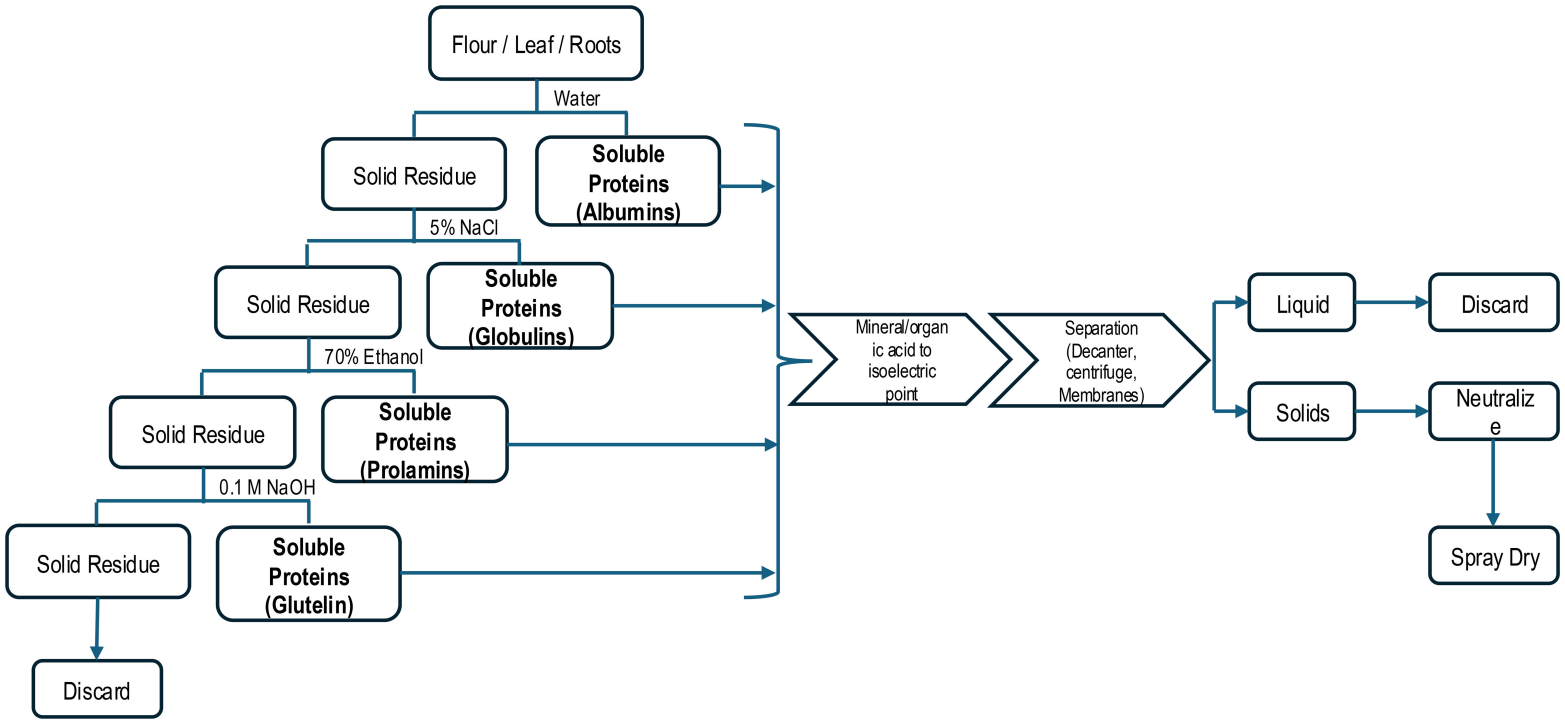
Osborne Extraction Protocol for Plant-based Proteins.

Numerous research papers have provided valuable information on total protein characterization and composition, functionality, and nutritional properties from products produced in laboratories using extraction methods not practical for commercialization. The so-called Osborne fractionation process relies on utilizing several different solvents (e.g. NaCl, ethanol, and low ionic strength NaOH) after an initial aqueous protein solubilization in stepwise manner to solubilize remaining proteins in a plant-based extraction (**Table 2**) (Dias et al., 2024; Osborne, 1924). This process is tedious and seldom used beyond research labs (**Figure 3**). However, the data it provides can be used to engineer commercial processes beyond the common alkalization and isoelectric precipitation method commercially used. The use of Osborne solvents enables extraction of specific solubility classes with highly specialized protein physical or physiological (bioactivity) functionality. Commercial products derived from these unique processes are usually low volume and more expensive to produce.

**Table 2:** Osborne protein composition in plants of commercial interest after extraction from plant-based components^1^.

Solvent	Protein classification	Grains (% by wgt.)^2^								Agricultural byproducts (% by wgt.)^2^			
		Soybeans	Wheat	Yellow pea	Oats	Brown rice	Faba bean	Maize (corn)	Barley	Wheat bran	Pea pods	Rice bran	Brewer’s grains
Water	Albumins	0.1-0.5	16-34	18-25	1-12	5-10	79.89	6.6-12.8	3-4	18-21	29.9-37.4	24-43	11
0.4 M NaCl	Globulins	80-90		55-65	70-80	7-17	21.7	7.0-9.1	10-20		16.1-19.1	13-36	15
70-90% Ethanol	Prolamins	1.2-1.4	48-62	4-5	4-15	3-6	3.45	22.2-47.5	35-45	0.75-1.5	6.7-13.8	1-5	35-55
0.1 M NaOH	Glutelins	Up to 11	15-31	3-4	<10	75-81	42.98	12.8-38.8	35-45	18-55	38.1-38.9	22-45	35-45
Total Protein		40	9.0-12.7	23.1-30.9	12-20	7.1-8.3	20-35	7.6-14.9	8-13	18	10-13	10-15	15-25
References		Nishinari et al., 2014	Schmid et al., 2017	Lam et al., 2018	Li et al., 2025	Amagliani et al., 2017	Das et al., 2023	Devi et al., 2024; Osborne, 1924	Linko et al., 1989	Sardari et al., 2019	Karabulut et al., 2023	Amagliani et al., 2017; Fabian and Ju, 2011	Devnani et al., 2023

^1^Extraction time and temperatures are typically 25° C or 50° C for a period of 1 hour.

^2^Percentages will not add up to 100% because actual values vary amongst varieties being extracted.

### Optimizing alkalization and acidification extraction

Alkali extraction is the most common method to extricate PBP by biomass cell wall disruption (Qiaoyun et al., 2017; Cui et al., 2020). Extraction efficiency is influenced by the biomass to solvent ratio, cation selection, ionic strength, temperature, time, and pH (Kumar et al., 2021; Sari et al., 2015). Elevated pH achieved by alkalizing agents like NaOH, KOH, and Na_2_CO_3_, begins solubilizing pectin in plant structure cell walls to release proteins, and subsequently breaking protein disulfide bonds to increase solubility in the extraction solvent (del Mar Contreras et al., 2019). Protein solubility increases at higher pH values as acidic and neutral amino acids become ionized. Extraction efficiency is optimized by many factors at this stage to optimize protein yield, maintain acceptable flavor and physical functionality, and minimize production costs. Several crops of commercial importance have well investigated factors manufacturers use to their advantage for optimized production (**Table 1**). Nuanced challenges are briefly discussed below.

### Particle size

Plant matrix particles procured from Extraction Stage 1 influence protein yield and extraction time. Increased surface area from smaller particles is better for interacting with extractant solvent and solubilizing protein from cell walls (Zhao et al., 2014). However, caution is advised when reducing particle size. Although the temptation is to produce a fine powder to maximize protein yield, heat generated during this process will denature proteins as noted earlier. Too fine of a powder will cause particle packing making it difficult to drain solvents, cause entrainment, and an increased likelihood of fines in finished products (Zhao et al., 2014). In theory, only outer cell layers are being milled to release their protein contents (Campbell and Glatz, 2009). Analytical instrumentation measuring particle size is misleading because it cannot differentiate between an intact cell mass and compartmentalized cell wall structures with partial protein extraction. Ideally extraction yields are achieved from particles with diameters equivalent to one or two cells from a particular crop.

### Solute:solvent ratio

The biomass to solvent ratio is carefully balanced to use a minimal solvent amount to accelerate protein diffusion. Excessive solvent is not only costly, but an imbalanced solvent to biomass ratio will limit driving forces extracting intracellular protein moving into solution. Currently biomass to solvent ratios of 1:50 are common in commercial operations to optimize maximum protein yields. The use of excessive solvent might yield more protein, but this benefit is easily offset by chemical costs and downstream waste mitigation expenses. A limited number of companies use countercurrent extraction methods, but the limited protein yield may negate a return on additional capital costs when constructing a facility (Lestari et al., 2010).

### Water chemistry

Many commercial protein factories use city water or their own well-water to extract PBP. In some cases, they “soften” their water in a demineralization process. However, not being diligent about recharging ion exchange chromatography resins could lead to challenges in protein extraction during alkalization steps including solubility reduction by forming protein/metal complexes that aggregate. Plant phytic acid accelerates complex formation. Beneficial to human consumption, peptide/zinc complexes are promoters of mineral absorption through the peptide transport mechanism for increased small intestine digestibility (Udechukwu et al., 2016). Faba bean proteins are metal ion chelators, and this impacts protein recovery in heterogeneous protein systems (Shi et al., 2024). Pumpkin seed protein peptides also have zinc-chelating properties that influence zinc bioaccessibility and bioavailability (Peng et al., 2022). Proteinates formed with PBP have low dissociation constants (high affinity). In fact, industrial usage of crude PBP extracts includes water remediation to coagulate metal ions from turbid water (Okoro et al., 2022). Chelated metal peptide complexes at low concentrations can be used advantageously in extraction as “dark mediators” to increase protein solubility subsequently improving nutritional and functional properties (Song, 2013).

### Alkalizing and acidifier cation

NaOH is the industry standard but from an environmental and human health perspective it is less desirable because of elevated Na^+^ levels in final products. KOH is an alternative given its similar chemical characteristics as NaOH. The mode of action for hydroxyl salts of Na^+^ and K^+^ is to solubilize proteins from plant cells by solubilizing the cellular membrane and concurrently breaking protein disulfide bonds responsible for stabilizing the protein’s internal structure (Asen et al., 2023; Berghout et al., 2015). Internal disruption and increases in electrostatic repulsion further rearrange polypeptide chains producing more secondary structures like β-turns and β-sheets (Zhu et al., 2021; Görgüç et al., 2020). At optimum pH levels, these actions enhance protein release, recovery, and yield. Additional studies are needed comparing extraction efficiency of Na^+^ versus K^+^ and the effect on protein functionality and sensorial properties. Recently, KOH and Ca(OH)_2_ salts have been proposed as alkalizing agents for leaf protein extraction. After extraction, the remnants can be recycled back on fields as fertilizers and are therefore environmentally better (Adamczyk et al., 2010). An additional benefit of less sodium in PBPC and PBPI would increase their use in healthy and nutritional foods. KOH is comparably efficient to NaOH in leaf protein yield and cost in use, but Ca(OH)_2_ is economically unfavorable in protein recovery despite the ability to recover heat from this reaction (Zhang et al., 2018). Protein extraction yield from oat grains was lower when using KOH for alkalization compared to NaOH (Dyshlyuk et al., 2017), although any differences in protein functionality between extraction methods were not reported. Functionalities of alkaline extracted yellow pea PI are reportedly influenced predominantly by cultivar (Cui et al., 2020). However, Chang et al. (2022) demonstrated functionality is affected using different cations. Whereas alkalized caseinate (sodium, calcium, and potassium) functionality has been thoroughly studied (Liao et al., 2022; Liu et al., 2024), studies on alkalizing cations effect on PBP physical functionality are still needed.

### Ionic strength and pH endpoints

Extraction yield and solubility correlates well with pH extremes (~pH 3 and ~pH 9) (Preece et al., 2017). In general, lower pH values promote protein particle aggregation for solid partitioning whereas alkaline pH values promote protein solubility by increasing electronegative charge on the protein surface during extraction. Briefly, chemical reactions occur at the amino acid level when carboxyl groups lose a proton as pH 9.0 is reached. At the acidification step, amino acid amide groups gain an extra proton making the protein molecule electropositive. When there is net zero charge (isoelectric point), proteins aggregate and precipitate from solution. Optimizing alkaline pH is crucial to avoid protein disaggregation and hydrolysis that impacts protein functionality (Choi et al., 2006; Renkema et al., 2000). Also, the effect of pH and temperature will cause potential amino acid racemization, crosslinking, loss of amino acids, and lysinoalanine formation that impacts protein digestibility (del Mar Contreras et al., 2019; Deleu et al., 2019). This is true for all crops selected for extraction. Oilseed crop defatted meals may have slightly lower protein yields because protein surface active properties form microemulsions within the aqueous solvent during extraction (Zhao et al., 2023). The driving forces for these interactions are hydrophobic amino acids in the protein’s primary sequence resulting in electrostatic interactions and hydrogen bonding between proteins and residual oils in the meals (Correa et al., 1998).

### Extraction temperature

Protein solubility decreases with increasing denaturation, which impacts finished protein physical properties. The goal is to use a temperature sufficient to accelerate protein extraction while stabilizing covalent interactions within protein structures during folding/unfolding (Sari et al., 2015; Zhang et al., 2015). Given that PBPC and PBPI are heterogeneous protein mixtures, extraction temperature should not exceed the lowest denaturation temperature of major protein groups in the target crop. However, some PBP companies advantageously use temperature when targeting specific functional properties for commercial proteins. Controlled heating of soy proteins improved several functionalities including foaming, gelation, emulsification, and fibrillation (Chen et al., 2025). Another consideration when selecting extraction temperatures is to minimize occurrences of undesirable enzymatic reactions. This can be difficult given plants grow over a wide temperature range. Good protein yield is observed at 25° C but it requires longer hold times (Zhang et al., 2014). Nonetheless, most laboratory and pilot plant PBP extractions use extraction temperatures of 50-60° C (Sari et al., 2015). This temperature range minimizes denaturation and is low enough to limit endogenous proteases from catalyzing protein hydrolysis during extraction. Although an alkaline pH would favor proteolytic activity, many commercialized plant-based enzymes require higher temperatures (60-75° C) for optimum hydrolysis (Troncoso et al., 2022). Further study is needed to determine if this is true for all endogenous botanical alkaline proteases.

### Exposure time

Protein solubilization and aggregation is a kinetically rapid reaction more dependent on level of protein denaturation than extraction temperature (Preece et al., 2017). Particle size is a controlling parameter, and finely ground flours will solubilize quicker because increased surface area is creating a shorter diffusion path. This reaction follows first-order kinetics (Preece et al., 2017). Many studies reported 1–2 hours extraction times (Sari et al., 2015). One consideration in the time-temperature relationship is that non-ruptured cells during extraction may still have metabolic activity. Plants experiencing stress can initiate programmed cell death and metabolically induce plant proteases capable of hydrolyzing extracted proteins (Cai et al., 2017). Therefore, it is best to identify a maximum temperature and minimal holding time in protein extraction optimization.

These extraction parameters and their details require attention in optimized process design for commercialized production of PBPC and PBPI at price points affordable for food scientists to formulate food products. Some highly specialized products with unique functional or nutritional properties can be prepared using modifications of the alkalization/isoelectric point process. Osborne compounds (e.g., NaCl) or solvents (e.g., saline or ethanol) can be added to the alkalizing and acidifying solvents but at much lower concentrations than specified in the Osborne fractionation process.

### NaCl extraction

NaCl protein extraction is practiced in biochemistry labs for many years to “salt-in” and “salt-out” proteins, and its use is still occasionally reported in the literature targeting difficult to extract proteins for improved functionality (Oroumei et al., 2024; Sun et al., 2022). Others have reported using low ionic strength NaCl in their alkalizing extraction solvent to improve seed storage protein solubility (Wanasundara et al., 2012). This method works well in laboratory and pilot plant facilities but is challenging at commercial scale. Corrosive properties of NaCl are detrimental to processing equipment and downstream solvent remediation is environmentally challenging and expensive. Nonetheless, several companies have further developed these methods for commercial advantage and launched PBPC and PBPI (Segali et al., 2024; Shi et al., 2023; Willemsen et al., 2024).

### Ethanol extraction

Much like NaCl, ethanol use in extracting proteins is best performed in laboratories or small, highly specialized manufacturing plants. Ethanol extraction at low concentrations is very efficient in fostering self-aggregates of many PBP incapable of being aqueously extracted. Often there are unique functionalities associated with these protein fractions. The key challenges of working with ethanol in a large facility include the licensing and record keeping requirements, and high costs associated with utilizing food grade absolute alcohol. Furthermore, extreme caution and vigilance must be maintained when working with flammable and explosive chemicals in a manufacturing environment. Small volume production of ethanolic (and NaCl) extracted proteins could find a niche in processing small volume supply chain materials of agricultural and food byproducts. Recently Privatti et al. (2024) reported saline and ethanol extraction of soy 7S proteins from okara, the solid byproduct of water-extracted soybean production, had excellent surface-active functional properties including water- and oil-holding capacity, emulsification, and foaming, but the protein preparation was insoluble in aqueous solutions. Alcohol-extracted proteins from BSG have high water holding capacity and gel formation despite poor aqueous solubility (Chin et al., 2024). These results were expected since BSG are byproducts from aqueous grain extraction. The alcohol soluble protein classes were mostly the storage proteins hordeins and glutelins from barley grain (Celus et al., 2006). Nanoparticles that can be used as fat mimetics are formed by reacting whey proteins with ethanol and NaCl followed by heat-setting the protein aggregates (Charitou and Moschakis, 2024). Protease hydrolysis of whey proteins in the presence of Mg^2+^ promotes self-assembly into nanotubes that are good vehicles for encapsulation and constructing fibrillated structures (Fuciños et al., 2021; Jiang et al., 2024).

The effects of ethanol on protein functionality result from conformational changes on solubilized and insoluble proteins. Aqueous ethanol solutions impact protein secondary and tertiary structure (Dufour and Haertle, 1990) causing denaturation (Herskovits and Mescanti, 1965; Mousavi et al., 2008) and reduction in solubility (Yoshikawa et al., 2012a, b; Yoshizawa et al., 2014)). Interestingly, susceptible protein molecules showed no increase in solubility mixed with lower ethanol concentrations indicating any favorable interaction with the hydrophobic amino acids was offset by the unfavorable hydrophilic amino acids (Yoshikawa et al., 2012a). As proteins unfold, the absence of water in a protein’s surrounding environment causes hydrophilic regions of the protein to form internal hydrogen bonds between them (Andreadis and Moschakis, 2023). The interaction between Gly, Asp, and His amino acid residues and ethanol is unfavorable, and results in free energy being transferred from the amino acids to ethanol wherein the entire protein molecule precipitates. Negative conformational changes in secondary and tertiary structures were reversible when proteins were re-solubilized in aqueous ethanol below 20% concentration (Dufour and Haertle, 1990).

### Extraction co-products

Rapidly growing plant cells synthesize between 100,000 and 1 million different molecules for a wide variety of functions and are often co-extracted with proteins (Fang et al., 2019). Many of these phytochemicals are secondary metabolites that serve physiological purposes within plants. For example, factors such as polyphenols, phytic acid, tannins, saponin, and enzyme inhibitors are synthesized to defend against pests, predators, and other harmful substances (Popova and Mihaylova, 2019). These phytochemicals do not affect the plant even when excessive amounts are formed in defense or by overzealous use of fertilizers and pesticides (Sinha and Khare, 2017). However, many of these phytochemicals are not metabolized by humans and many animals and thus are labeled as antinutritional factors (Samtiya et al., 2020). There are two primary modes of action for antinutritional factors. Nutrients must be bioaccessible and bioavailable to be utilized by any species consuming them from their diet (Grace et al., 2022). Some plants produce flavanols and tannins that are digestive enzyme inhibitors impeding starch and protein digestion (Barrett et al., 2018). Oxalic acid and phytic acid form insoluble mineral complexes with proteins making them non-absorbable in the gastrointestinal tract (Borquaye et al., 2017; Gupta et al., 2013). Many antinutritional factors are heat labile and destroyed if grain is cooked, but moist heat must rupture cell membranes to release antinutrients and expose them to heat for destruction (Faizal et al., 2023). However, phytic acid or phytate found predominantly in bran is heat stable, and its presence should be minimized if the grains were milled and the bran removed. However, most flours used for protein extraction are whole grain, and the mineral/protein complexes do impact protein recovery and nutritional value. Mineral deficiencies in older humans, pregnant women, and young children have traditionally been treated with inorganic mineral supplements. Over consumption of foods with inorganic minerals for long periods of time can be toxic. Foods fortified with mineral/protein complexes are emerging as an alternative to inorganic mineral supplementation to reduce mineral malnourishment without toxic side effects (Jindal et al, 2023).

Polyphenolic compounds often co-extract with proteins resulting in both desirable and undesirable dark colors and bitter flavor notes but they also have positive physiological effects especially as antioxidants. Few studies have reported the impact of protein/phytic acid complexes on protein functionalities other than decreased protein solubility, but gelation, foaming, and emulsification would likely be impacted (Amat et al., 2023). Other polyphenols cause conformational changes allowing conjugation with proteins to alter protein surface hydrophobicity, charge, and interfacial properties (Manzoor et al., 2024; Saffarionpour, 2025). These interactions have positive consequences on emulsifying and gelling properties. Additionally, they have a bioactive role as powerful antioxidants. Removal of dark colored polyphenols is challenging, but limited success has been found with silica and styrene resins, diatomaceous earth, and slightly acidified NaCl solutions (Pickardt et al., 2015).

## Co-extraction of plant and animal proteins

Global protein demand has surged over 40% the last two decades because of increasing population, a larger percentage of an aging population intent on consuming animal protein, global affluency, and per capita consumption (Smith et al., 2024). Two-thirds of global protein consumption is from meat, fish, poultry, and dairy; grains are the leading source of PBP. Furthermore, ABP are promoted as having higher nutritional qualities than PBP including essential amino acid content, better digestibility, and delivery of physiologically important nutrients (e.g. Fe^2+^ and Ca^2+^) (Day et al., 2022). Continued stresses on Earth’s biosphere from animal agriculture indicates a novel strategy must be developed to meet future protein needs. Livestock and grain for the foreseeable future will be the primary dietary sources to meet protein demand. Culture and familiarization drive many of our dietary habits, and modern man has adapted to animal-based products. The dilemma for mankind becomes how to provide more ABP outside of raising and processing whole animals.

The precedence for co-extraction of ABP and PBP has been established by the biopharma industry by recovering therapeutic proteins from plants grown in CEA facilities. However, their objective was focused on recovery of specific protein molecules as pharmacological agents, and the presence of other plant proteins were a nuisance that required extensive processing steps to devoid their preparations of these and other “contaminants”. A limited number of publications have discussed these challenges and techniques used to isolate targeted proteins and their challenges with downstream processing. Most concluded emerging MF technologies would be less challenging and less expensive than precision fermentation (Basaran and Rodríguez-Cerezo, 2008; Buyel et al., 2015; Chaudhary et al., 2024).

The strategy to co-isolate ABP and PBP simultaneously to capitalize on beneficial nutritional and functional properties has been accomplished by blending and co-precipitating/co-aggregating protein concentrate and isolate powders to create mixed protein powders (Alu’datt et al., 2012; Kristensen et al., 2020, 2021; Pan and Zhong, 2016). There are many negative factors associated with this approach, but three are especially important keeping in mind cost of use and environmental impact. There is a limited selection of ABP; dairy and egg are the primary sources and powdered meat concentrates have not established much of a supply chain. Commercial PBPC, PBPI, ABPC, and ABPI are expensive, and re-processing them would add further cost. Furthermore, there will be losses of some proteins from incompatibility during processing and protein yields will be reduced. Re-processing blended proteins requires using harsh chemicals again and potentially impacting protein structure, but also environmentally it is an unsound process.

**Figure 4:**
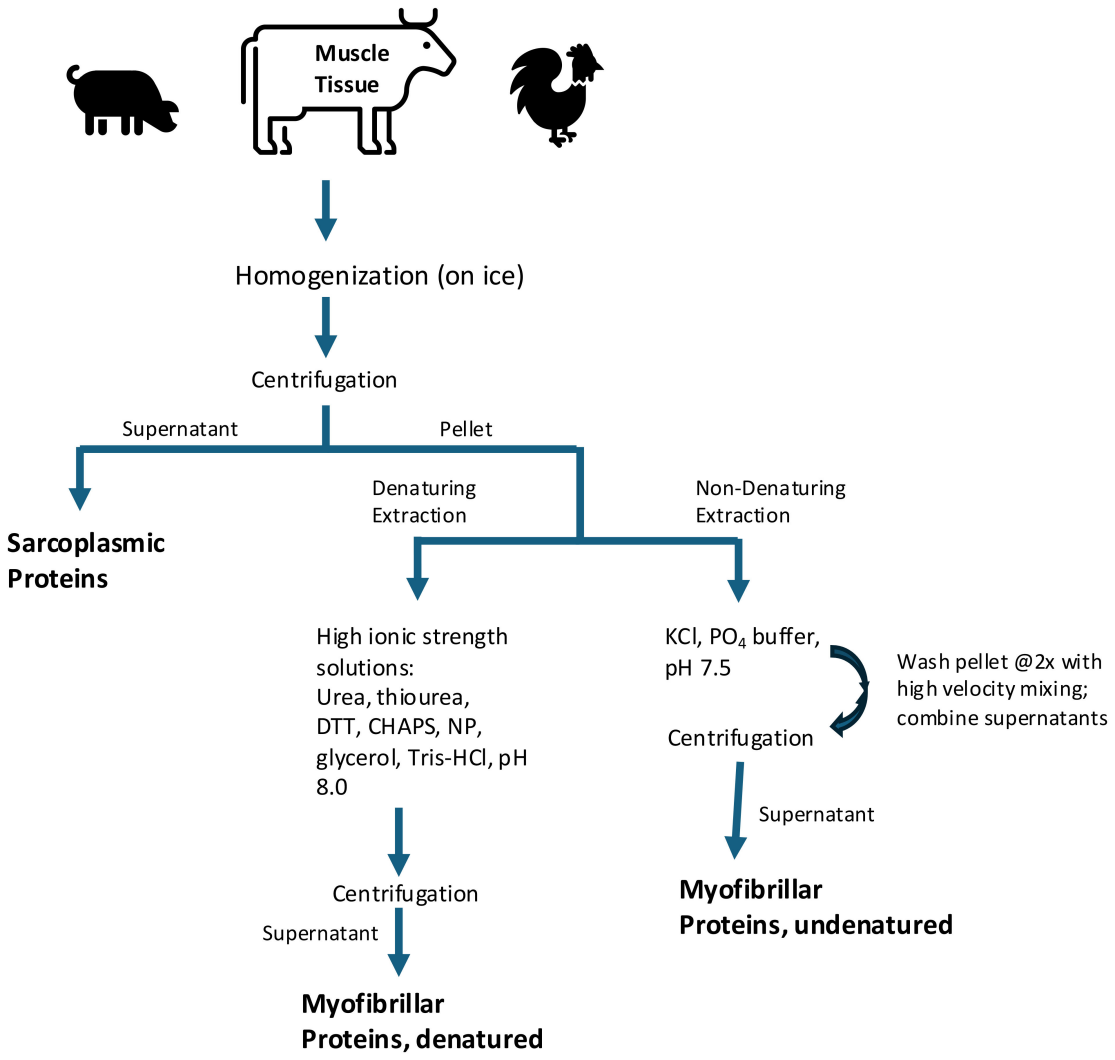
Exemplary Extraction of Animal Muscle Proteins^1,2^. ^1^Multiple muscle protein extraction protocols have been published for use in laboratories. Commercial processes have been proprietary. ^2^Reference: modified from della Malva et al. (2018).

In recent years there has been interest in inserting animal genes into plants for expression of ABP molecules that can be co-extracted with endogenous plant proteins (Long et al., 2022). This novel approach has not been attempted by many research laboratories or commercial organizations for food ingredient production. Furthermore ABP, like PBP, are complex mixtures of expressed proteins making downstream processing and concentration a challenge (**Table 3**). ABP are often much larger molecules that are synthesized, structured, and compartmentalized in discrete fluids (e.g. milk and egg yolk) or complex structures (e.g. muscle fibers) that are easier to utilize as raw materials for extraction (**Figure 4**). Commercially utilized methods described earlier in this publication (**Figure 2**) would need further investigation into their potential to extract and concentrate commercial quantities of proteins to be manufactured into proteinaceous food ingredients. Historical downstream processing of targeted therapeutic proteins has involved using traditional biopharma developed methodology. Although these methods enable highly purified products, it is inconceivable to utilize these methods in a commodity manufacturing environment and provide enough protein to meet global demand at reasonable price expectations. Another important consideration will be consumer-acceptance of non-traditional protein sources as demonstrated by consumer reluctance to consume insect and cell-cultivated protein products (Smith et al., 2024).

**Table 3:** Animal protein candidates for transgenic insertion into plants used for co-protein extraction^1^.

Source	Protein	Mol. Wgt. (KDa)	# Disulfide bonds/Free thiol groups	Surface hydrophobicity	Molecular conformation	pI	Functional properties^2^	T_d_^3^ (°C)	Refs^4^
Cow Milk	ß-lactoglobulin	18.3	2/1	hydrophobic	Central beta barrel formed by 8 antiparallel β-strands	5.2	E,F,G	70-80	Milk
	Lactoferrin	78		hydrophilic	2 symmetric, globular lobes connected by a short α-helix	8.0	AO,B,FeB,IM	90	
	Bovine serum albumin	66.5	17/1	hydrophilic	Single polypeptide chain, organized into 3 homologous domains, each containing 2 subdomains	5.3	AO,TP,B	65	
	ß-casein	23.6	0/2	amphiphilic	Random coil; asymmetric structure; polar N-terminal region; less polar C-terminal region	4.6	E,F,G,B	70-80	
Chicken Egg	Ovalbumin	45	1/4	amphiphilic	Serpin structure with 3 α-helices and 3 β-sheets	4.5	E,F,G,AO	80	Eggs
	Ovotransferrin	76	15/2	hydrophilic	2 Globular lobes; linked by α-helix	6.1	AO,B,FeB,IM	61-84	
	Ovomucoid	28	9/0	hydrophilic & hydrophobic regions	3 Tandem domains in primary sequence with 9 intra-domain S-S bonds and 5 carbohydrate side chains	4.1	AO,B,FeB	95	
Bovine Steer	Myosin	223	0/2	hydrophilic & hydrophobic regions	Hexameric protein with 2 heavy chains with large, coiled structures; 2 pairs of light chains	5.4	FB,T,WB	50	Meat
	Hemoglobulin	64.5	0/0	hydrophilic	Tetrameric protein (4 subunits); 2 α and 2 β chains; heme group in each subunit	7.1	OB	60-85	
	Myogloblin	17	0/2	hydrophilic	Globular, single polypeptide chain folds into 8 α -helices and non-covalently binds a heme group	7.1	OB	60	
	Collagen	300	17/0	hydrophobic	Right-handed triple helix composed of 3 left-handed polypeptide chains; high # of Pro & OH-Pro residues	8.0	T,B	65	

^1^Only major proteins of commercial relevance from each source are listed.

^2^Functionality abbreviations: AO, antioxidant; B, bioactivity; E, emulsifying; F, foaming; FB, fat binding; FeB, iron binding; G, gelling; IM, immunomodulatory; OB, oxygen binding; T, texturizing; TP, transport protein; WB, water binding.

^3^Td: Thermal denaturation temperature.

^4^References: Milk: Dyrda-Terniuk and Pomastoski (2023); Farrell et al. (2004); Mulvihill and Donovan (1987); Eggs: Doi and Kitabatake (1997); Rossi and Schiraidi (1992); Rostamabadi et al. (2023); Meat: Xiong et al. (1987); Xiong (1997).

Many different plant species can be considered as production hosts including cereal, oil, and legume seeds, leafy crops, fruits, vegetables, algae, mosses, and aquatic plants (Twyman et al., 2003). In essence, plants are inexpensive bioreactors that are highly scalable –the technology needed to plant, cultivate, and harvest plants has been utilized by several generations of farmers. Capital and running costs for protein production are significantly lower in plants. The production cost of producing egg white avidin in transgenic maize plants is 200% less expensive than isolating it from egg whites (0.5% of the egg-based cost) (Hood et al., 2002). Furthermore, plant cell biology can closely mimic animal cellular traffic and post-translationally modify animal proteins with sugars, phosphorus, ubiquitin, and lipids. Gene expression and post-translational modification have been successful in therapeutic proteins and there is little compelling evidence to suggest this would be any different for functional proteins.

Seed-bearing plants, including soybean, wheat, rice, pea, chickpea, faba beans, and lentils, are currently commercially exploited for PBP extraction. Their ability to store protein within the endosperm or cotyledon provides many advantages for localization of recombinant animal proteins without compromising molecular encoding and translation of PBP molecules (Khan et al., 2012). Protein expression and stability in plants is influenced by genotype selection, transgene copy number and zygosity, and construct design. There are also intangible properties of seeds making them an ideal vehicle to deliver native PBP and transgenic ABP when processed. Recombinant protein stability after extended storage has been established (Nochi et al., 2007; Stõger et al., 2000).

Tuber plants, particularly potatoes and yams, should also be considered as target hosts for transgenic transformation especially if the structure and function of inserted proteins could be dependent on post-translational phosphorylation (Bernal et al., 2019). Tubers have the same function as seeds for storing proteins until they are needed to support plant reproduction, but they can additionally execute an eloquent series of reactions between protein kinases, phosphatases, and phytohormones to phosphorylate storage proteins (Umezawa et al., 2013). Phosphorylated proteins are important in protein aggregation and influencing protein secondary structure (Che et al., 2025). Peptides produced by *in vivo* proteolysis of phosphorylated proteins also have a physiological role in essential mineral uptake for human nutrition (Tenenbaum et al., 2022). Dairy casein proteins are a good source of phosphorylated proteins.

## Molecular farming for novel food protein combinations

Transgenesis of ABP genes into plants could potentially increase protein nutritional quality when co-extracted with PBP. Furthermore, physical functional properties could possibly be improved or present additional or unforeseen functionalities in subsequent hybrid PBPC and PBPI. Several criteria should be considered when deciding which protein genes to insert in a plant’s genomic repertoire. The primary decision is which nutritional, physical functionality, sensorial, and/or physiological (bioactive) properties will be targeted for protein product improvement. This is often prescribed by the availability of target gene sequences and its ease of insertion into a production plant host (Gerszberg and Hnatuszko-Konka, 2022; Tusé et al., 2024). Genetic stability of the inserted genes as well as expression of allergens and anti-nutritional factors will need to be monitored. Potential impact on protein synthesis in the host plant including ability to be successfully cultivated, ease of protein extraction with minimal deviation from typical processing methods, impact on overall protein yield and composition, and loss of other potential functionalities will need to be investigated. Finally, it will be important to monitor any environmental impact from transgenic plants. The biopharmaceutical industry estimated downstream processing (extraction, concentration, and drying) accounted for 45 to 90% of the manufacturing process costs (Owczarek et al., 2019). Food protein manufacturing should be at the lower end of these estimates. Ultimately, successful gene expression and synthesis of novel, functional proteins that can be economically extracted from plant material is necessary to compete against currently marketed PBPC and PBPI.

**Figure 5:**
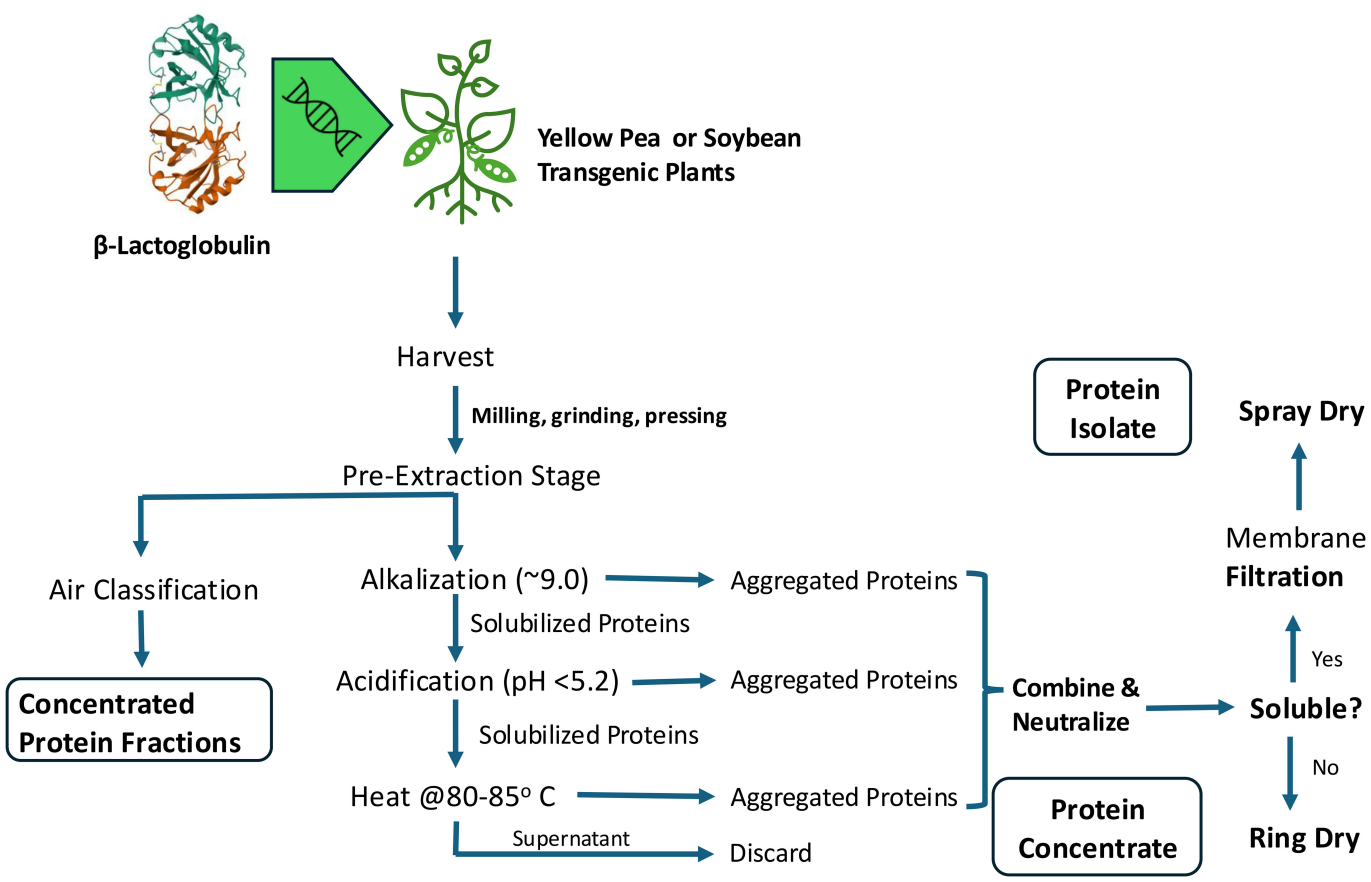
Potential Commercial Process Flow for Co-Extraction of Animal- and Plant-Based Proteins from Transgenic Plants.

These decisions should enable a well-designed schematic to commercially produce nutritional and functional protein products. Individual proteins from meat, dairy, and eggs would be the likely candidates given the large amounts of technical information available about these proteins. These protein classes account for most of the environmental stress from animal production. Studies on the nutritional and functionality observations acquired by simply blending or co-precipitating ABP and PBP will provide insights into potential challenges that will be encountered when extracting proteins from these transgenic plants. However, there are only a limited number of publications in these areas, and most of the work was done using blended laboratory produced or commercially available protein preparations (Alu’datt et al., 2012; Kristensen et al., 2020, 2021). Plants are intricate systems and potential impacts on plant growth and yield from transgenic animal gene insertion are not well understood yet. However, designing a process for co-extraction of animal and plant proteins should consider aligning their physical properties (**Tables 1**, **3**). For example, equivalent molecular conformations, isoelectric points, and thermal denaturation might improve extraction efficiency. Slight differences in PBP extraction methods will be necessary to adequately extract both protein sets with minimal damage to nutritional value and functionality (**Figure 5**). These nuances should be considered when developing optimized extraction protocols for commercial production of hybrid protein concentrates and isolates. Extensive characterization of resulting products must be conducted. A thorough understanding of the nutritional and safety equivalence is imperative.

We have proposed a generalized process to co-extract ABP and PBP from transgenic plants (**Figure 5**). Other techniques and physical methods were minimally discussed, but there would be value to increase extraction complexity by use of enzymes and other emerging technologies developed at commercial scale. For example, pulsed electric field and ultrasound have shown promise in PBP extraction, and these technologies might be useful for mixed systems (Karabulut et al., 2023; Kumar et al., 2021; Zhao et al., 2023).

## Summary and future research necessities

Much information in this review focused on challenges of PBP extraction because there is limited information on extracting proteins from plants with ABP transgenes. The exception is a few studies on challenges of extracting pharmacologically important compounds, but methods used in initial plant cell disassembly are significantly different from commercially used methods for PBP concentrates and isolates manufacture. Nevertheless, hybrid concentrates and isolates of animal proteins being transgenically expressed in plants and co-isolated with plant proteins could yield an innovative subset of alternative protein products. Improved nutritional and functional properties would be primary targets, but in theory an added benefit would be less environmental impact for meeting future animal protein demands. However, there is a great deal of research ahead for society to confirm these theories. Gene expression and protein synthesis in the host plant system will need verification. Protein extraction from plant-based materials is challenging, and the same tools used for PBP may have to be modified or improved upon to ascertain commercially viable yields and cost parity, nutritional equivalence, and food ingredient safety standards. Outside of the extraction challenges and knowledge about current PBP systems, efforts must be initiated to construct databases on transgenic hybrid products. Some key, but not inclusive, factors are understanding impact on crop agronomic factors, genetic containment, protein/protein interactions, allergenic effects, functional properties, and sensorial properties. Previous studies from blending animal and plant protein powders frequently described the behavior of combined proteins during and/or after heating (Alu’datt et al., 2012; Kristensen et al., 2020, 2021). As described in this review, PBP molecules are very reactive at the molecular level to thermal exposure. Choices are plentiful of ABP genes to insert into plants, but many of the blended investigations were done with dairy proteins, more specifically whey proteins, limiting precedent data on meat and egg co-precipitate products.

Finally, multiple inflection points are converging in our global boundaries including environmental impacts and changes, a growing and aging population, increased global protein consumption, and animal welfare concerns. These criticalities foreshadow an imperative need for communication with multiple actors in the food supply chain, including regulatory and policy makers, to educate them about the value of introducing this technology and these products mainstream. There will undoubtedly be public and legislative resistance, but urgency to answer the many questions needs to be recognized in preparation for rapid consumer protein demand within the next two decades.
